# Management protocol for emergency aneurysm craniotomy clipping in non-major COVID-19 epidemic areas in Beijing, China

**DOI:** 10.1186/s41016-020-00217-x

**Published:** 2020-12-22

**Authors:** Yu Chen, Xiaolin Chen, Li Ma, Xiaofeng Deng, Zelin Li, Xun Ye, Hao Wang, Shuai Kang, Yan Zhang, Rong Wang, Dong Zhang, Yong Cao, Yuanli Zhao, Shuo Wang, Jizong Zhao

**Affiliations:** 1 Department of Neurosurgery, Beijing Tiantan Hospital, Capital Medical University, Beijing, 100070 China; 2 Department of Neurosurgery, Peking University International Hospital, Peking University, Beijing, China; 3 China National Clinical Research Center for Neurological Diseases, Beijing, China; 4 Stroke Center, Beijing Institute for Brain Disorders, Beijing, China; 5 Beijing Key Laboratory of Translational Medicine for Cerebrovascular Disease, Beijing, China; 6 Beijing Translational Engineering Enter for 3D Printer in Clinical Neuroscience, Beijing, China

**Keywords:** COVID-19, Aneurysmal subarachnoid hemorrhage, Non-major epidemic areas, Craniotomy clipping

## Abstract

**Background:**

An epidemic of COVID-19 broke out in Wuhan, China, since December 2019. The ordinary medical services were hindered. However, the emergency cases, including aneurysmal subarachnoid hemorrhage (aSAH), still required timely intervention. Thus, it provoked challenges to the routine management protocol. In this study, we summarized our experience in the emergency management of aSAH (Beijing Tiantan Protocol, BTP) in Beijing, China.

**Methods:**

Demographic, clinical, and imaging data of consecutive emergency aSAH patients who underwent craniotomy clipping during the COVID-19 epidemic season were reviewed and compared with the retrospective period last year. Subgroup analysis was further performed to assess the outcomes of different screening results and several detailed protocols. Neurological outcomes were evaluated by the modified Rankin Scale (mRS).

**Results:**

A total of 127 aSAH were referred to our emergency department, and 42 (33.1%) underwent craniotomy clipping between January 20, 2020, and March 25, 2020. The incidence of preoperative hospitalized adverse events and the perioperative outcomes were similar (− 0.1, 95% CI − 1.0 to 0.8, *P* = 0.779) to the retrospective period last year (January 2019–March 2019). After the propensity score matching (PSM), there were still no statistical differences in prognostic parameters between the two groups. Eight (19.0%) of the 42 individuals were initially screened as preliminary undetermined COVID-19 cases, in which 2 of them underwent craniotomy clipping in the negative pressure operating room (OR). The prognosis of patients with varied COVID-19 screening results was similar (*F*(2, 39) = 0.393, *P* = 0.678). Since February 28, 12 cases (28.6%) received COVID-19 nucleic acid testing (NAT) upon admission, and all showed negative. The false-negative rate was 0.0%. The preoperative hospitalized adverse events and postoperative prognosis were still similar between patients with and without COVID-19 NAT (− 0.3, 95% CI − 1.4 to 0.9, *P* = 0.653).

**Conclusions:**

Our emergency surgery management protocol (BTP) is reliable for scheduling emergency aneurysm craniotomy clipping in non-major epidemic areas.

## Background

In December 2019, an outbreak of pneumonia with unknown causes broke out in Wuhan, China. On January 7, 2020, Chinese scientists isolated a new type of coronavirus from virus-infected pneumonia patients [1]. It was later designated as coronavirus disease 2019 (COVID-19) by the World Health Organization (WHO) in February 2020 [2]. As of March 28, 2020, more than 500,000 COVID-19 pneumonia cases have been confirmed globally, with a mortality rate of 4.3%.

On January 20, 2020, the first COVID-19 patient in Beijing (China) was confirmed in our hospital. During the past 65 days, a total of 416 confirmed cases were detected in Beijing. Beijing has a permanent population of 21.53 million, according to statistics from the Beijing Municipal Bureau of Statistics in 2019. Aneurysmal subarachnoid hemorrhage (aSAH) has an approximate incidence of 9 per 100,000 and a mortality rate of about 60% within 6 months [3]. It means that a large number of aSAH patients were diagnosed every day. Beijing Tiantan Hospital is the biggest neurosurgical center in China, and most aSAH patients in Beijing were treated in our hospital during this particular COVID-19 epidemic season. To our knowledge, no previous studies have proposed a standardized and highly reliable emergency aSAH surgical treatment protocol in non-major COVID-19 epidemic areas. We summed up our experience in the safe and effective protocol (Beijing Tiantan Protocol, BTP) of emergency admissions, transfer, surgery, and ward management for aSAH in non-major COVID-19 epidemic areas (416 to 21.53 million), based on the published incident management system by our hospital [4]. It can provide references for most non-major epidemic cities in the world.

## Methods

### Study design and participants

This retrospective study included a single-center consecutive cohort of emergency aSAH inpatients from Beijing Tiantan Hospital (Beijing, China) between January 20, 2020, and March 25, 2020. The inclusion criteria were as follows: (1) patients’ preoperative CTA or digital subtraction angiography (DSA) indicated that SAH’s responsible lesion was cerebral aneurysms, (2) patients undergoing emergency craniotomy clipping for the responsible aneurysms, (3) the duration between emergency admission and a craniotomy was less than 72 h. Written informed consent for collecting clinical information was obtained from each patient at admission. According to the Declaration of Helsinki guidelines, this study was performed and was approved by the Institutional Review Board at Beijing Tiantan Hospital.

Finally, a total of 42 aSAH patients were enrolled in this study. To assess whether the BTP increased the risk of preoperative hospitalized adverse events and poor postoperative prognosis, we collected the baseline characteristics and prognostic data of emergency aSAH patients during the retrospective period last year (18 cases).

### Data collection and definition

Epidemiological, demographic, clinical, laboratory, treatment, and outcome data were extracted from electronic medical records using a standardized data collection form. Fever was defined as an axillary temperature of at least 37.3 °C [5]. Positive epidemiological history, laboratory tests, and imaging findings were determined according to the Chinese management guideline for COVID-19 (version 5.0) [6]. Fisher scale, modified Fisher scale, Hunt-Hess scale, World Federation of Neurological Surgeons (WFNS) scale, and Glasgow Coma Scale (GCS) were used to evaluate the categories of severity [7–10]. DCI was defined as new cerebral infarction identified on CT or magnetic resonance imaging (MRI), or proven at autopsy after excluding procedure-related infarction, or a new focal neurologic deficit and a persistent clinical deterioration after excluding other potential causes [11, 12]. The mRS score was obtained when discharged. All data were checked by two physicians (YC and XLC), and a third researcher (LM) adjudicated any difference in interpretation between the two primary reviewers.

### Beijing Tiantan Protocol

**Fig. 1:**
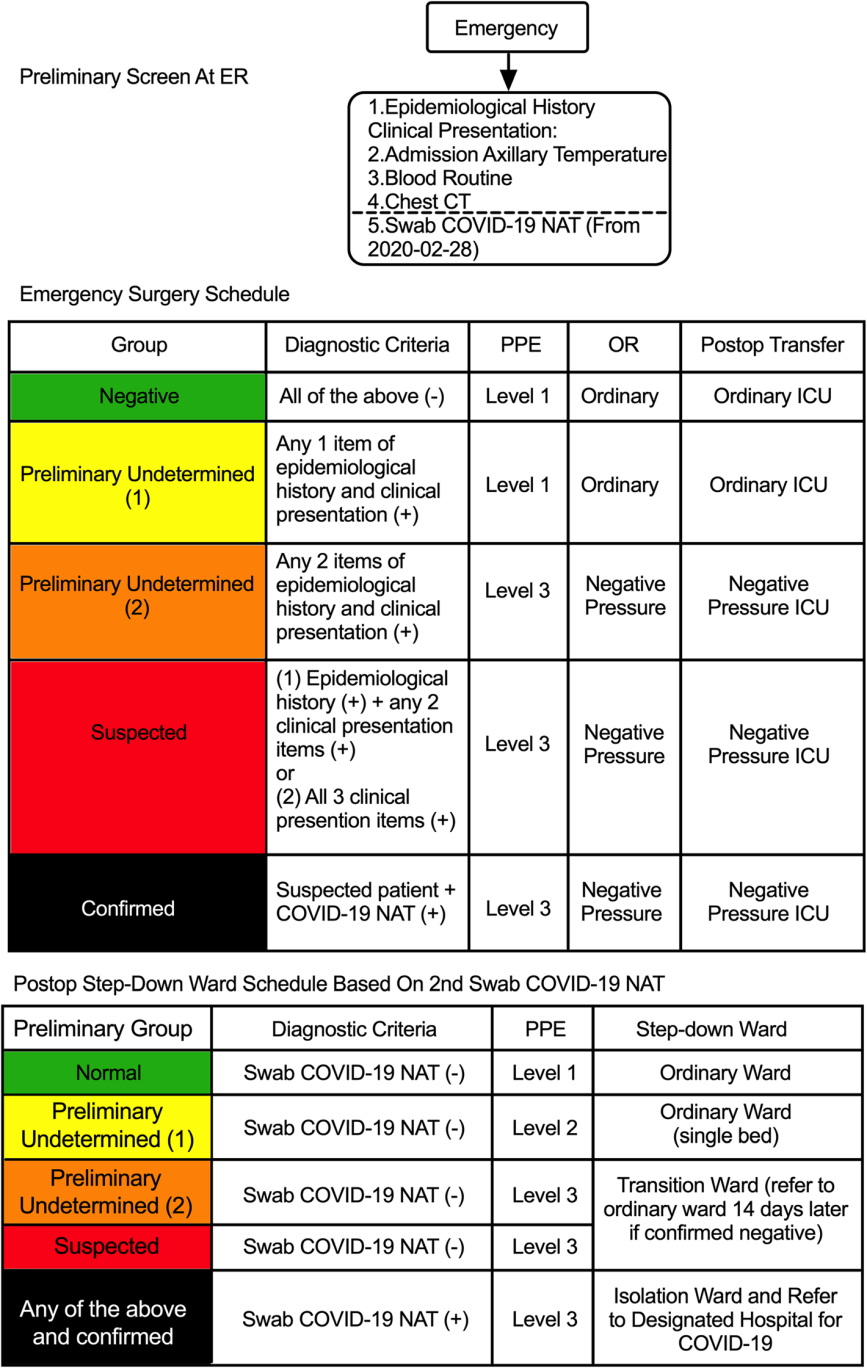
Beijing Tiantan Protocol for aSAH during the COVID-19 outbreak

After the patient came to the emergency department, the emergency physicians prescribed CT/CTA and clinical presentations to confirm the diagnosis of aSAH. Emergency physicians protect themselves according to the second-level protection standards, which is work clothing, shoe covers, disposable working caps, N95 masks (replace every 4 h or when it is wet or at any time if there is pollution), goggles, protective clothing or isolation clothes, and latex gloves [6, 13]. Preliminary screening for COVID-19 in the emergency department included epidemiological history, admission axillary temperature, routine blood test, and lung CT [6]. And then, the consultation team of COVID-19 prevention and control experts (consist of directors of the emergency department, respiratory department, and nosocomial infection department) in our hospital would evaluate the preliminary screening results and divided them into five groups: negative, preliminary undetermined (1), preliminary undetermined (2), suspected, and confirmed (Fig. 1).

Since February 28, COVID-19 NAT was added to the screening protocol. The first COVID-19 NAT (pharyngeal swab, result waiting time 6 h) and routine preoperative preparation would be performed immediately in the transition ward (the standard of protection is the same as COVID-19 isolation ward: third-level protection standards). If the consultation result was “suspected or confirmed patient,” we do not recommend delaying surgery if the hospital has adequate protective equipment. We have dedicated patient transport channels, dedicated surgical teams, and dedicated negative pressure OR. The surgical team should protect themselves according to the third-level protection standards: work clothing, protective boot covers, disposable working caps, N95 masks, goggles, medical protective masks (or positive pressure head covers), protective clothing, double-layer latex gloves [6, 13]. After the surgery, the patients would be sent to the COVID-19 isolation negative pressure ICU. For the “preliminary undetermined” patients, if any two epidemiological history and clinical presentation items were positive, the management protocol would be similar to the “suspected and confirmed” cases. If any 1 item of epidemiological history and clinical presentation was positive, the treatment protocol would be similar to the screening “negative” cases. For the screening “negative” patient, emergency craniotomy for clipping aneurysms would be arranged in the conventional OR. Both of the screening “negative and preliminary undetermined (1)” patients would be sent to the ordinary ICU after the surgery. The second COVID-19 NAT would be performed 24 h after the first NAT for all emergency aSAH patients. The patient would be transferred to the ordinary ward/ordinary ward (single bed)/transition ward (refer to ordinary ward 14 days later if confirmed negative)/isolation ward (refer to a designated hospital for COVID-19 when suitable), according to the second COVID-19 NAT.

Perioperative management of aSAH was the same as that recommended in previous guidelines [3, 14]. The screening procedures were performed again in the patients with postoperative fever of unknown causes and preoperative undetermined suspected cases. If the COVID-19 NAT were positive, the patients would be transferred to the COVID-19 designated hospital for further treatment after the intracranial condition stabilized. For the screening negative patients, paramedic adopted first-level protection standards (first-layer work clothes [e.g., scrubs], disposable working caps, surgical masks) after the operation. For the preliminary undetermined (1) patients, the second-level protection standards were adopted. Third-level protection standards were used for preliminary undetermined (2)/suspected/confirmed patients after the operation [6, 13]. Family members of the patient are not allowed to accompany the patient in the ward during the hospitalization. Visitors need to pass the preliminary screening of epidemiological history and temperature measurement.

### Statistical analysis

Continuous and categorical variables were presented as mean ± standard deviations (SD) and counts (with percentages). Two-tailed *t* tests were used otherwise for the continuous variable with Gaussian distribution. The Mann-Whitney *U* (Wilcoxon) test was used to compare non-normal distribution continuous variables. For categorical variables, either the Fisher exact test or the Pearson chi-square test was used. We further conducted a propensity score analysis methodology (PSM) to compare the outcomes. Based on the covariates from the logistic model, each patient’s propensity score with respect to each baseline characteristic was generated, including demographic, clinical, and radiographic characteristics. A nearest matching algorithm with a 1:1 ratio was applied. The outcomes of interest were preoperative adverse events during hospitalization, postoperative complications, and discharge mRS score. Statistical analysis was performed using SPSS (Version 25.0, IBM). The significance threshold was set at a 2-sided *P* value less than 0.05.

## Results

### Baseline characteristics

**Fig. 2:**
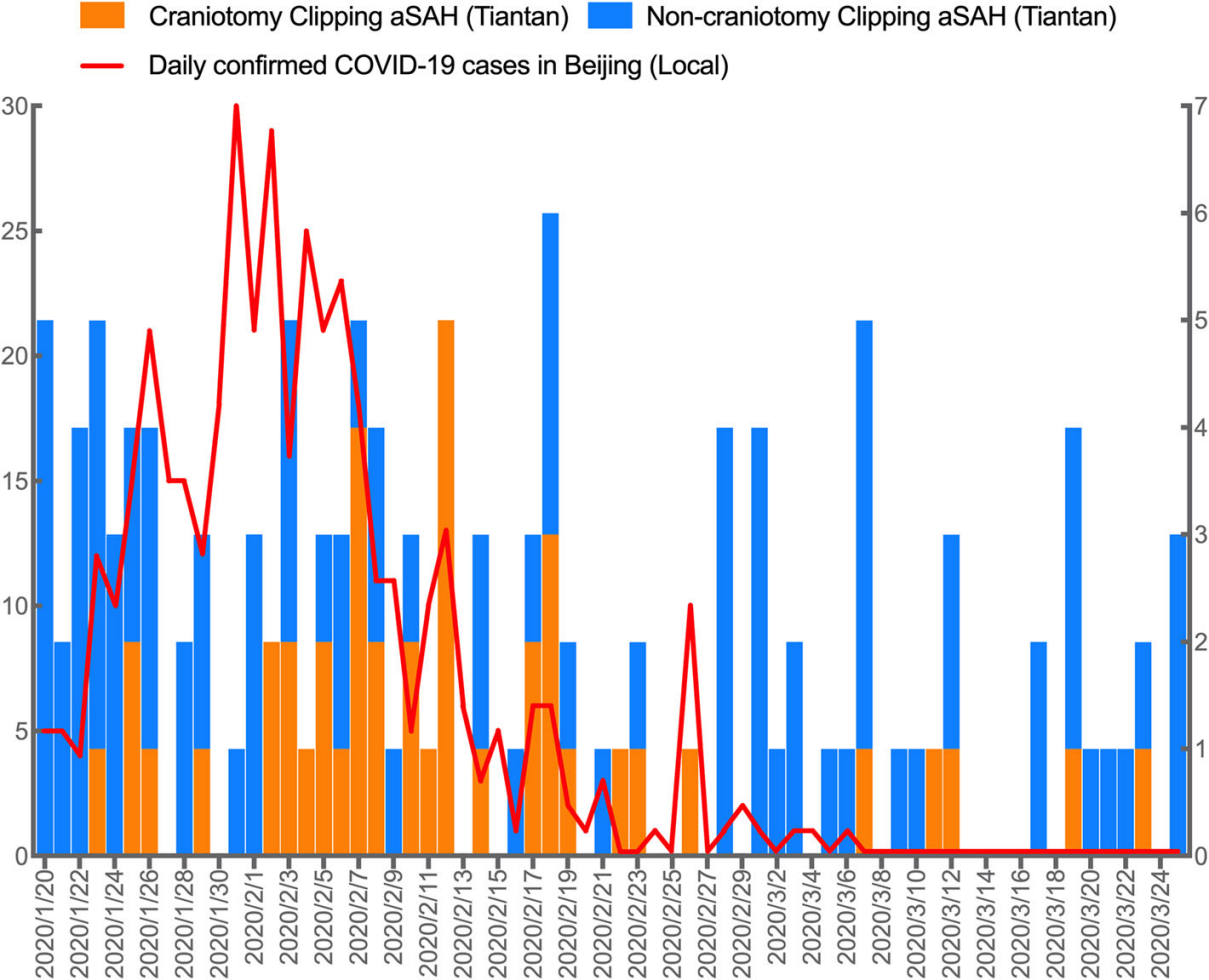
Emergency aSAH in our hospital at different stages of the epidemic

From January 20, 2020, to March 25, 2020, nine patients were diagnosed with COVID-19 with positive nucleic acid testing (NAT) in our hospital for all kinds of emergency patients. A total of 127 aSAH patients referred to our emergency department, and 42 (33.1%) of them underwent craniotomy clipping (Fig. 2). The patients’ mean age was 54.9 ± 8.6 years (range 35–74 years) (Table 1). Eight cases (19.0%) were initially screened as preliminary undetermined COVID-19 cases, and 2 of them underwent craniotomy clipping surgery in the negative pressure operating room (OR). Since February 28, 12 cases (28.6%) received COVID-19 NAT upon admission and showed negative results, and the false-negative rate was 0.0%. The location of responsible aneurysms was mostly in the anterior communicating artery (ACoA, 31.0%), posterior communicating artery (PCoA, 35.7%), and middle cerebral artery (MCA, 26.2%). The modified Fisher grade and Hunt-Hess grade was 3.0 ± 1.1 and 1.9 ± 0.5, respectively. The duration between admission and craniotomy was 34.8 ± 18.5 h. Generalized epilepsy during hospitalization before craniotomy occurred in two patients (4.8%).

**Table 1:** Comparison of emergency aSAH patients between the current period and the respective time period last year

Characteristics	Current	Last year	*P* value^a^
No. of patients	42	18	
No. of aneurysms	45	19	
Sex (male)	24 (57.1)	8 (44.4)	0.366
Age, years	54.9 ± 8.6	49.4 ± 10.7	0.039*
Admission stratification			
Normal patient	34 (81.0)		
Mild suspected patient	8 (19.0)		
Suspected patient	0 (0.0)		
Confirmed patient	0 (0.0)		
COVID-19 NAT (*n* = 12) (negative)	12 (100.0)		
False negative COVID-19 NAT	0 (0.0)		
Location of responsible aneurysm			
ACoA	13 (31.0)	7 (38.9)	0.585
PCoA	15 (35.7)	5 (27.8)	
ACA	2 (4.8)	0 (0.0)	
MCA	11 (26.2)	6 (33.3)	
Others	1 (2.4)	0 (0.0)	
Fisher grade	3.1 ± 1.1	2.9 ± 1.0	0.555
Modified Fisher grade	3.0 ± 1.1	3.1 ± 1.1	0.835
Hunt-Hess grade	1.9 ± 0.5	2.4 ± 0.6	0.003*
WFNS grade	2.7 ± 0.9	2.8 ± 1.1	0.690
GCS	12.9 ± 1.2	12.0 ± 2.3	0.367
Duration between rupture and craniotomy, hours	71.1 ± 55.2	40.7 ± 45.6	0.044*
Duration between admission and craniotomy, hours	37.1 ± 16.8	28.7 ± 11.7	0.058
Preoperative adverse events during hospitalization			
Rebleeding	0 (0.0)	0 (0.0)	> 0.99
Infarction	0 (0.0)	0 (0.0)	> 0.99
Epilepsy	2 (4.8)	1 (5.6)	> 0.99
Duration of surgery, h	3.5 ± 1.0	4.1 ± 2.3	0.277
Intraoperative blood loss, ml	260.7 ± 238.0	258.3 ± 152.7	0.969
Mean hospital LOS, days	14.9 ± 8.8	17.4 ± 6.9	0.298
Hospitalization cost, dollars^b^	13385.6 ± 6973.4	14006.1 ± 5640.4	0.740
Postoperative complications			
Postoperative hemorrhage	0 (0.0)	1 (5.6)	0.660
Postoperative DCI	13 (31.0)	8 (44.4)	0.315
Postoperative DVT	10 (23.8)	1 (5.6)	0.190
Postoperative intracranial infection	5 (11.9)	4 (22.2)	0.528
Discharge mRS score			
Mean	1.9 ± 1.6	2.1 ± 1.6	0.779
0–2	29 (69.0)	12 (66.7)	0.856
3–6	13 (31.0)	6 (33.3)	

Values are numbers of cases (%) unless otherwise indicated. Mean values are presented with SDs

*ACA* anterior cerebral artery, *ACoA* anterior communicating aneurysm, *aSAH* aneurysmal subarachnoid hemorrhage, *COVID-19* coronavirus disease 2019, *CT* computed tomography, *DCI* delayed cerebral ischemia, *DVT* deep vein thrombosis, *GCS* Glasgow Coma Scale, *LOS* length of stay, *MCA* middle cerebral artery, *mRS* modified Rankin Scale, *NAT* nucleic acid testing, *PCA* posterior cerebral artery, *WFNS* World Federation of Neurological Surgeons

^a^*P* values indicate differences between the current period and the respective time period, and *P* less than 0.05 was considered statistically significant

^b^The exchange rate between RMB and USD is 1 USD = 7.0942 RMB

*Statistical significance (*p* < 0.05)

### Comparison between current and retrospective period last year

Eighteen emergency aSAH patients underwent craniotomy clipping in our hospital during the retrospective period last year (Table 1). Compared with them, the patients who received BTP were older (5.5, 95% confidence intervals [CI] 0.3 to 10.7, *P* = 0.039) and had lower Hunt-Hess grades (0.5, 95% CI 0.2 to 0.8, *P* = 0.003). There are no statistical differences in the duration between admission and craniotomy between the two groups (37.1 ± 16.8 vs. 28.7 ± 11.7; 8.4, 95% CI − 0.3 to 17.1, *P* = 0.058). It indicates that BTP may not prolong the duration of preoperative hospitalization. Finally, the incidences of preoperative hospitalized adverse events and postoperative neurofunctional outcomes (− 0.1, 95% CI − 1.0 to 0.8, *P* = 0.779) were similar between the two groups. Postoperative delayed cerebral ischemia (DCI) (0.6, 95% CI 0.2 to 1.7, *P* = 0.315) occurred in thirteen cases (31.0%) in the BTP group and 8 cases (44.4%) in the control group. According to propensity scores, we matched 9 cases with BTP to 9 cases last year (Table 2). The two groups were compared to verify that there are no significant differences in baseline characteristics after the PSM. Finally, we found that there were no significant differences in preoperative adverse events during hospitalization (*P* > 0.99), postoperative complications (*P* > 0.99), and discharge mRS score (0.8, 95% CI − 1.9 to 1.5, *P* = 0.788).

**Table 2:** Comparison of emergency aSAH patients between the current period and the respective time period last year after propensity score matching (PSM)

Characteristics	Current	Last year	*P* value^a^
No. of patients	9	9	
No. of aneurysms	10	10	
Sex (male)	5 (55.6)	4 (44.4)	> 0.99
Age, years	50.11 ± 9.3	45.3 ± 11.3	0.342
Admission stratification			
Normal patient	6 (66.7)		
Mild suspected patient	3 (33.3)		
Suspected patient	0 (0.0)		
Confirmed patient	0 (0.0)		
COVID-19 NAT (*n* = 2) (negative)	2 (100.0)		
False negative COVID-19 NAT	0 (0.0)		
Location of responsible aneurysm			
ACoA	3 (33.3)	3 (33.3)	0.842
PCoA	3 (33.3)	2 (22.2)	
ACA	0 (0.0)	0 (0.0)	
MCA	3 (33.3)	4 (44.4)	
Others	0 (0.0)	0 (0.0)	
Fisher grade	3.0 ± 1.2	2.9 ± 1.1	0.839
Modified Fisher grade	3.2 ± 1.0	2.7 ± 1.2	0.302
Hunt-Hess grade	2.2 ± 0.4	2.2 ± 0.7	> 0.99
WFNS grade	2.4 ± 0.9	2.6 ± 1.2	0.819
GCS	12.6 ± 1.8	12.3 ± 2.3	0.825
Duration between rupture and craniotomy, h	56.9 ± 52.8	56.2 ± 60.5	0.980
Duration between admission and craniotomy, h	35.6 ± 15.3	31.9 ± 12.3	0.577
Preoperative adverse events during hospitalization			
Rebleeding	0 (0.0)	0 (0.0)	> 0.99
Infarction	0 (0.0)	0 (0.0)	> 0.99
Epilepsy	0 (0.0)	0 (0.0)	> 0.99
Duration of surgery, h	3.7 ± 1.1	4.9 ± 2.9	0.264
Intraoperative blood loss, ml	188.9 ± 126.9	266.7 ± 188.7	0.320
Mean hospital LOS, days	17.4 ± 13.9	17.9 ± 9.0	0.937
Hospitalization cost, dollars^b^	17,354.9 ± 12,110.1	13,147.1 ± 4200.9	0.339
Postoperative complications			
Postoperative hemorrhage	0 (0.0)	0 (0.0)	> 0.99
Postoperative DCI	4 (44.4)	4 (44.4)	> 0.99
Postoperative DVT	3 (33.3)	1 (11.1)	> 0.99
Postoperative intracranial infection	2 (22.2)	2 (22.2)	> 0.99
Discharge mRS score			
Mean	1.8 ± 1.7	2.0 ± 1.7	0.788
0–2	7 (77.8)	6 (66.7)	> 0.99
3–6	2 (22.2)	3 (33.3)	

Values are numbers of cases (%) unless otherwise indicated. Mean values are presented with SDs

*ACA* anterior cerebral artery, *ACoA* anterior communicating aneurysm, *aSAH* aneurysmal subarachnoid hemorrhage, *COVID-19* coronavirus disease 2019, *CT* computed tomography, *DCI* delayed cerebral ischemia, *DVT* deep vein thrombosis, *GCS* Glasgow Coma Scale, *LOS* length of stay, *MCA* middle cerebral artery, *mRS* modified Rankin Scale, *NAT* nucleic acid testing, *PCA* posterior cerebral artery, *WFNS* World Federation of Neurological Surgeons

^a^*P* values indicate differences between the current period and the respective time period, and *P* less than 0.05 was considered statistically significant

^b^The exchange rate between RMB and USD is 1 USD = 7.0942 RMB

### Subgroup analysis

There were no statistical differences in prognosis between the negative cases and preliminary undetermined COVID-19 cases (1)/(2) (*F*(2, 39) = 0.393, *P* = 0.678) (Table 3). Although patients undergoing COVID-19 NAT took slightly longer time from admission to a craniotomy (35.9 ± 15.3 vs. 40.2 ± 20.4 h; 4.4, 95% CI − 7.3 to 16.0, *P* = 0.453), there were no differences in preoperative hospitalized adverse events and postoperative prognosis of the patients with or without COVID-19 NAT (− 0.3, 95% CI − 1.4 to 0.9, *P* = 0.653, Table 4).

**Table 3:** Subgroup comparison of different preliminary COVID-19 screening results

Characteristics	Normal (conventional OR)	Mild suspected (conventional OR)	Mild suspected (negative pressure OR)	*P* value^a^
No. of patients	34	6	2	
Duration between admission and craniotomy, hours	38.5 ± 17.6	32.8 ± 7.4	25.5 ± 23.3	0.456
Preoperative adverse events during hospitalization				
Rebleeding	0 (0.0)	0 (0.0)	0 (0.0)	> 0.99
Infarction	0 (0.0)	0 (0.0)	0 (0.0)	> 0.99
Epilepsy	1 (2.9)	1 (16.7)	0 (0.0)	0.438
Duration of surgery, h	3.4 ± 0.9	3.3 ± 0.9	5.5 ± 0.0	0.007*
Intraoperative blood loss, ml	233.8 ± 136.9	200.0 ± 154.9	900 ± 848.5	< 0.001*
Mean hospital LOS, days	13.8 ± 7.3	19.3 ± 15.6	20.5 ± 0.7	0.250
Hospitalization cost, dollars^b^	12,695.9 ± 5226.2	17,568.7 ± 10,863.7	12,560.6 ± 397.0	0.290
Postoperative complications				
Postoperative hemorrhage	0 (0.0)	0 (0.0)	0 (0.0)	> 0.99
Postoperative DCI	9 (26.5)	3 (50.0)	1 (50.0)	0.453
Postoperative DVT	9 (26.5)	1 (16.7)	0 (0.0)	0.497
Postoperative intracranial infection	3 (8.8)	2 (33.3)	0 (0.0)	0.255
Discharge mRS score				
Mean	1.9 ± 1.6	2.2 ± 1.7	1.0 ± 1.4	0.678
0–2	23 (67.6)	4 (66.7)	2 (100.0)	0.466
3–6	11 (32.4)	2 (33.3)	0 (0.0)	

Values are numbers of cases (%) unless otherwise indicated. Mean values are presented with SDs

*aSAH* aneurysmal subarachnoid hemorrhage, *COVID-19* coronavirus disease 2019, *DCI* delayed cerebral ischemia, *DVT* deep vein thrombosis, *LOS* length of stay, *mRS* modified Rankin Scale, *OR* operating room

^a^*P* values indicate differences among different preliminary COVID-19 screening result, and *P* less than 0.05 was considered statistically significant

^b^The exchange rate between RMB and USD is 1 USD = 7.0942 RMB

*Statistical significance (*p* < 0.05)

**Table 4:** Comparison of emergency aSAH patients with/without COVID-19 NAT

Characteristics	Absent	Accept	*P* value^a^
No. of patients	30	12	
No. of aneurysms	32	13	
Sex (male)	16 (53.3)	8 (66.7)	0.430
Age, years	55.1 ± 8.3	54.5 ± 9.5	0.840
Admission stratification			
Normal patient	26 (86.7)	8 (66.7)	0.151
Mild suspected patient	4 (13.3)	4 (33.3)	
Suspected patient	0 (0.0)	0 (0.0)	
Confirmed patient	0 (0.0)	0 (0.0)	
Location of responsible aneurysm			
ACoA	8 (26.7)	5 (41.7)	0.675
PCoA	11 (36.7)	4 (33.3)	
ACA	1 (3.3)	1 (8.3)	
MCA	9 (30.0)	2 (16.7)	
Others	1 (3.3)	0 (0.0)	
Fisher grade	3.2 ± 1.0	2.9 ± 1.2	0.442
Modified Fisher grade	3.2 ± 1.1	2.8 ± 1.1	0.264
Hunt-Hess grade	1.9 ± 0.5	1.9 ± 0.5	0.928
WFNS grade	2.7 ± 0.9	2.8 ± 1.0	0.601
GCS	12.8 ± 1.3	13.1 ± 1.0	0.550
Duration between rupture and craniotomy, h	71.7 ± 55.3	70.0 ± 57.4	0.931
Duration between admission and craniotomy, h	35.9 ± 15.3	40.2 ± 20.4	0.453
Preoperative hospitalized adverse events			
Rebleeding	0 (0.0)	0 (0.0)	> 0.99
Infarction	0 (0.0)	0 (0.0)	> 0.99
Epilepsy	1 (3.3)	1 (8.3)	0.513
Duration of surgery, h	3.5 ± 0.9	3.5 ± 1.2	0.913
Intraoperative blood loss, ml	236.7 ± 130.6	320.8 ± 400.8	0.490
Mean hospital LOS, days	14.4 ± 9.7	16.3 ± 6.4	0.547
Hospitalization cost, dollars^b^	14,268.4 ± 7811.7	11,178.5 ± 3596.8	0.198
Postoperative complications			
Postoperative hemorrhage	0 (0.0)	0 (0.0)	> 0.99
Postoperative DCI	9 (30.0)	4 (33.3)	0.833
Postoperative DVT	8 (26.7)	2 (16.7)	0.481
Postoperative intracranial infection	3 (10.0)	2 (16.7)	0.558
Discharge mRS score			
Mean	2.0 ± 1.6	1.8 ± 1.5	0.653
0–2	20 (66.7)	9 (75.0)	0.874
3–6	10 (33.3)	3 (25.0)	

Values are numbers of cases (%) unless otherwise indicated. Mean values are presented with SDs

*aSAH* aneurysmal subarachnoid hemorrhage, *COVID-19* coronavirus disease 2019, *DCI* delayed cerebral ischemia, *DVT* deep vein thrombosis, *LOS* length of stay, *mRS* modified Rankin Scale, *NAT* nucleic acid test

^a^*P* values indicate differences between patients with or without COVID-19 NAT, and *P* less than 0.05 was considered statistically significant

^b^The exchange rate between RMB and USD is 1 USD = 7.0942 RMB

### Illustration case

**Fig. 3:**
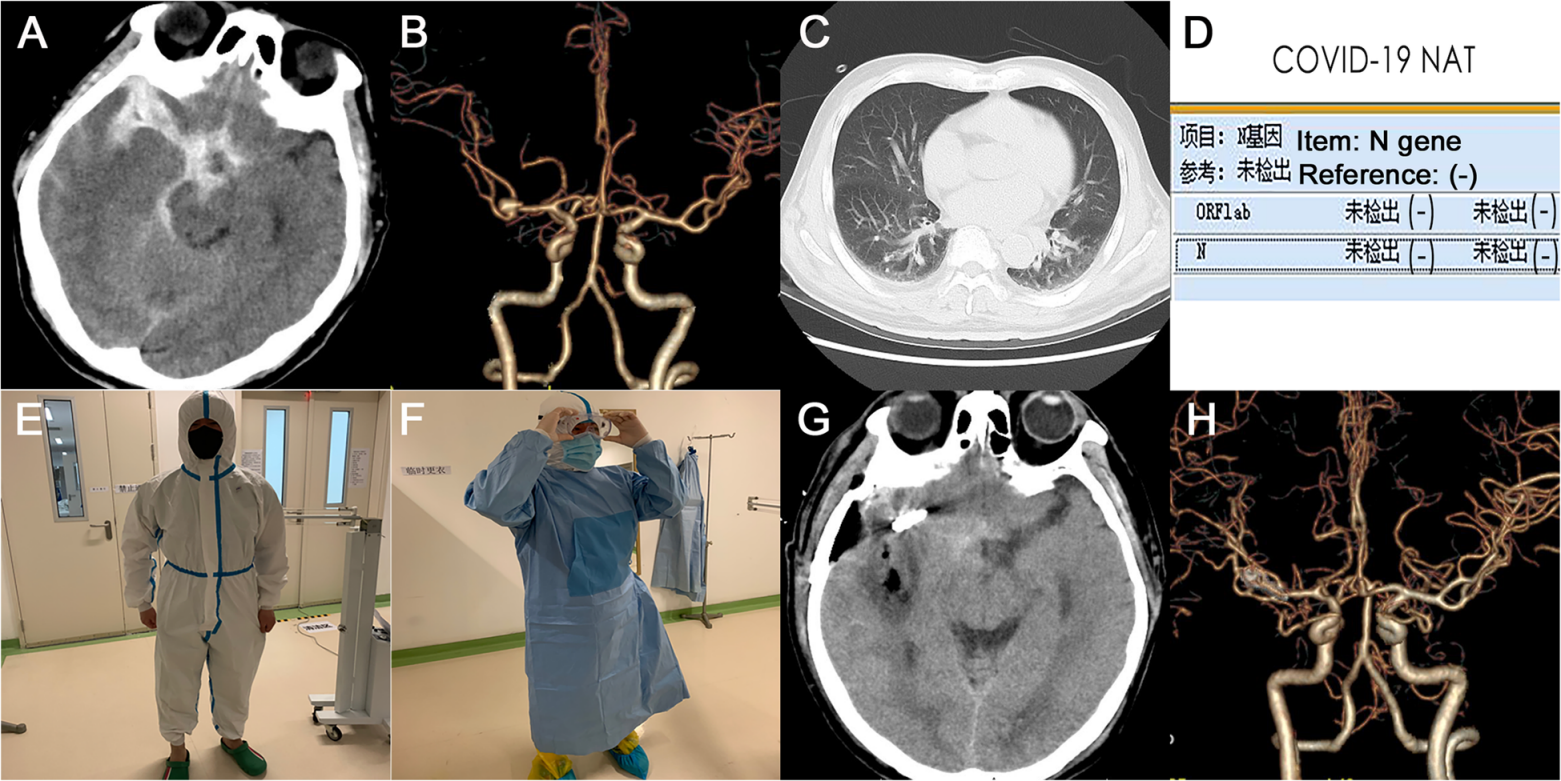
Illustration case

A 61-year-old male presented with sudden severe headache for 23 h with nausea and vomiting. Preoperative computed tomography angiography (CTA) revealed a right MCA M1 bifurcation irregular aneurysm (Fig. 3). The modified Fisher grade was four, and the Hunt-Hess grade was 2. The preliminary COVID-19 screening suggested positive axillary temperature (38.4 °C), positive lung CT, and negative COVID-19 NAT (once), and was classified as a preliminary undetermined (2) patient. The consultation group of COVID-19 prevention and control experts judged that the operation should be carried out in the negative pressure OR. The surgical team protected themselves according to the third level of protection standards. The operation was performed by a senior neurosurgeon (XLC) with 15 years of experience in aneurysm clipping. During the operation, the view field in the microscope was limited due to the fogging goggles. The operation duration was 5.5 h. Intraoperative blood loss was about 300 ml. The patient was transferred to the isolation negative pressure intensive care unit (ICU) after the operation, and the reexamination of COVID-19 NAT was still negative 24 h after the first NAT. He was then transferred to the ordinary neurosurgical ward (single bed) on the second postoperative day. No postoperative complications occurred, and the patient was discharged on the 7th postoperative day, with a modified Rankin Scale (mRS) score of 0.

## Discussion

COVID-19 was identified as a Public Health Emergency of International Concern (PHEIC) by the WHO in February 2020. A recent retrospective clinical study suggested that the nosocomial infection rate of COVID-19 was up to 41% [15]. It means that the front-line medical workers carry a considerable risk of infection of COVID-19 at work, especially in the absence of medical protection supplies. ASAH is a significant cause of morbidity and mortality throughout the world, which was reported a quarter of patients die, and rebleeding events occurred in 4–13.6% within the first 24 h [14, 16]. Therefore, in this special period, implementing the “safe rescue principle” diagnosis and treatment for emergency aSAH patients with limited medical resources is particularly important [17, 18]. After 2 months of practice and revision, we believe that our admission protocol (BTP) can complete the surgical treatment for emergency aSAH on the premise of ensuring safety in non-main COVID-19 epidemic areas.

Compared with the retrospective period last year, the number of emergency aSAH patients significantly increased, and the preoperative Hunt-Hess grade significantly decreased. It might be due to the emergency department’s closure in some other hospitals, resulting in more mild aSAH patients transported to our tertiary neurosurgical center. There is currently no evidence to confirm whether there is a correlation between aSAH and SARS-CoV-2.

Previous studies have indicated that COVID-19 could cause neurological disorders [19, 20]. For the central nervous system, patients with COVID-19 infection are at increased risk for ischemic stroke due to the increased D-dimer [6, 21]. Severe thrombocytopenia and increased blood pressure (BP) often occurs in critical patients of COVID-19, which might be responsible for the subsequent intracranial hemorrhage (ICH) and SAH. The Chinese Center for Disease Control (CDC) reported a case of COVID-19 whose cerebrospinal fluid (CSF) gene sequencing confirmed the presence of SARS-CoV-2.

In this protocol, we set up a definition of a quarantine transition ward for the COVID-19 epidemic. Patients before craniotomy were admitted to the transitional ward. If NAT’s reexamination is negative/positive, the patient would be transferred to a specialist ordinary ward or isolation ward after the surgery, respectively. The transitional ward’s existence enabled us to efficiently complete the preoperative preparation of emergency patients under the premise of safety. The establishment of specialized surgical transport channels, neurosurgeons, anesthesiologists, instrument nurses, itinerant nurses, and operating rooms is also crucial [18].

The current surge in suspicious infections worldwide has resulted in a shortage of COVID-19 NAT reagents, so whether NAT is needed to guide further treatment is an urgent question faced by most non-major epidemic areas. Due to the lack of COVID-19 NAT reagents at the early stage of the epidemic, our screening protocol did not include COVID-19 NAT. Since February 28, COVID-19 NAT was added to our screening protocol with a sufficient supply of NAT reagents. In this study, all 12 patients who underwent preoperative COVID-19 NAT were negative, with a false-negative rate of 0.0%. All patients in this cohort were excluded as preliminary undetermined cases upon discharge. Therefore, with strong epidemic prevention and control measures (such as screening of population epidemiological history, timely isolation of close contacts, adequate protective materials, and reduction of population movement), non-designated COVID-19 hospitals will have fewer opportunities to treat infected patients with aSAH in non-major epidemic areas. Therefore, excessive panic is unnecessary, and it may be more critical to enhance the clinician’s confidence and personal hygiene awareness and adequate protective materials. Besides, we found no significant differences in preoperative hospitalized adverse events and postoperative prognosis of the patients with or without COVID-19 NAT, although patients undergoing COVID-19 NAT required a slightly longer time from admission to a craniotomy (no statistical differences). Therefore, we believed that in a non-major epidemic city (416 to 21.53 million) lacking COVID-19 NAT reagents, it is advisable to adopt a screening method combining epidemiological history with clinical characteristics. However, we should be vigilant for false-negative COVID-19 NAT and asymptomatic infections. It was reported that the positive detection rate of COVID-19 NAT was thought to be only 30–50% [13]. About 30–60% of COVID-19 asymptomatic infections may exist in the current population [22]. The asymptomatic infections’ virus load is similar to symptomatic patients [23]. However, the implicit transmission ability of asymptomatic infections is still uncertain [24].

In this study, two patients were operated on in the negative pressure OR, during which the neurosurgeon followed the highest protection standards. However, the protective mask and the OR’s negative pressure often make the operators feel challenging to breathe. The goggles are easy to fog, which makes the operator’s vision under the microscope very fuzzy and narrow. Therefore, we recommended experienced senior neurosurgeons to perform the surgery. Otherwise, endovascular intervention is preferred. However, the high incidence of DCI after endovascular intervention should not be underestimated [25].

COVID-19 pneumonia is a massive challenge for all of humanity, and we hope that our research will serve as a reference for neurosurgical centers around the world. There are some limitations to this study. First, there are no confirmed COVID-19 patients in this study, so the screening protocol’s effectiveness cannot be verified. However, this protocol is based on the diagnostic criteria recommended by the CDC in Wuhan, China, which has been verified by a large number of patients, so we think this protocol is referential. Second, less operational experience in the negative pressure OR may not provide a reference for craniotomy in patients with confirmed COVID-19.

## Conclusions

Our study suggests that BTP could effectively complete the admission screening and surgical treatment of emergency aSAH patients in non-major COVID-19 epidemic areas. It may be used as a reference in local neurosurgical practices.

## Data Availability

All data relevant to the study are included in the article or uploaded as supplementary information.

## Abbreviations

ACoA: Anterior communicating artery
aSAH: Aneurysmal subarachnoid hemorrhage
BP: Blood pressure
BTP: Beijing Tiantan Hospital
CDC: Center for Disease Control
CI: Confidence intervals
CTA: Computed tomography angiography
CSF: Cerebrospinal fluid
DCI: Delayed cerebral ischemia
DSA: Digital subtraction angiography
ER: Emergency room
GCS: Glasgow Coma Scale
ICH: Intracranial hemorrhage
ICU: Intensive care unit
MCA: Middle cerebral artery
mRS: Modified Rankin Scale
NAT: Nucleic acid testing
OR: Operating room
PCoA: Posterior communicating artery
PHEIC: Public Health Emergency of International Concern
PPE: Personal protective equipment
SD: Standard deviations
WFNS: World Federation of Neurological Surgeons
WHO: World Health Organization
